# Large-scale live imaging of adult neural stem cells in their endogenous niche

**DOI:** 10.1242/dev.123018

**Published:** 2015-10-15

**Authors:** Nicolas Dray, Sébastien Bedu, Nelly Vuillemin, Alessandro Alunni, Marion Coolen, Monika Krecsmarik, Willy Supatto, Emmanuel Beaurepaire, Laure Bally-Cuif

**Affiliations:** 1 Paris-Saclay Institute for Neuroscience, CNRS UMR9197 – Université Paris-Sud, Team Zebrafish Neurogenetics, Avenue de la Terrasse, Building 5, Gif-sur-Yvette F-91198, France; 2 Laboratory for Optics and Biosciences, École Polytechnique, Centre National de la Recherche Scientifique (UMR 7645) and Institut National de la Santé et de la Recherche Médicale (U1182), Palaiseau 91128, France

**Keywords:** SHG/THG, Live imaging, Multiphoton microscopy, Neural stem cell, Pallium, Zebrafish

## Abstract

Live imaging of adult neural stem cells (aNSCs) *in vivo* is a technical challenge in the vertebrate brain. Here, we achieve long-term imaging of the adult zebrafish telencephalic neurogenic niche and track a population of >1000 aNSCs over weeks, by taking advantage of fish transparency at near-infrared wavelengths and of intrinsic multiphoton landmarks. This methodology enables us to describe the frequency, distribution and modes of aNSCs divisions across the entire germinal zone of the adult pallium, and to highlight regional differences in these parameters.

## Abstract (precis)

**Summary:** This methodology permits long-term imaging and tracking of NSCs in the adult zebrafish telencephalic neurogenic niche, allowing the frequency, distribution and modes of NSC divisions to be analyzed.

## INTRODUCTION

The vertebrate adult brain harbors restricted sites of constitutive neurogenesis, where neurons of high physiological impact are generated from adult neural stem cells (aNSCs). The molecular and cellular processes controlling aNSC maintenance and recruitment are incompletely understood, and the dynamics of their homeostasis at the population level in germinal niches remain largely unknown. This is largely due to two inherent properties of aNSCs that complicate their study *in vivo*. First, aNSC populations are largely quiescent, hence population dynamics are slow: exiting the quiescent state towards activation and division takes days or weeks (Kriegstein and Alvarez-Buylla, 2009; Morshead et al., 1994). Short-term live imaging over a couple of days at most is possible on cultured brain slices, whereas neurospheres or primary cultures allow longer term imaging, but the speed and modes of cell division are obviously modified under these non-physiological conditions (Pastrana et al., 2011). Second, the properties and dynamics of aNSC populations are intimately linked with physiological, mechanical or molecular input from the surrounding environment (the so-called ‘niche factors’), such as contact with blood vessels, the cerebrospinal fluid or other local NSCs, progenitors or neurons (Silva-Vargas et al., 2013). None of these elements can be reliably reconstituted *in vitro*.

**Fig. 1.**
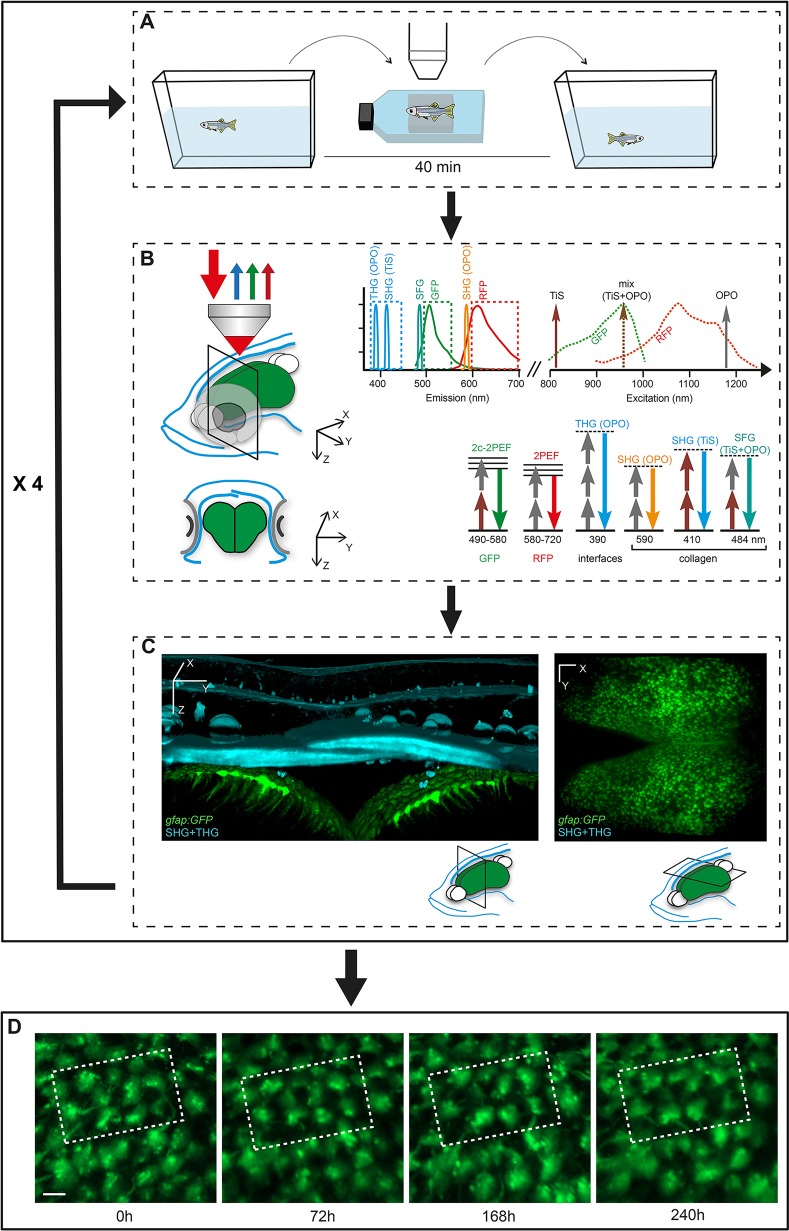
**Workflow for the tracking of zebrafish pallial aNSCs in their endogenous niche over weeks.** (A) The transparent double mutant fish line *casper*, crossed into fluorescent GFP and/or RFP backgrounds highlighting aNSCs and/or cell division events, is subjected to reiterated imaging at intervals of a few days over a period of weeks (see Fig. S2). The whole procedure (anesthesia, positioning and imaging) takes around 40 min. (B) Combined multicolor fluorescence/harmonics multiphoton microscopy performed *in vivo* through the skin and skull (blue on the schematized fish head, left panel) down to the pallium (green) (top: lateral 3D view, bottom: cross section at the indicated plane) permits the concomitant recording of aNSCs (GFP, green arrows; RFP, red arrows) and skin/skull structures (SHG/THG harmonics, blue arrows). The combination of TiS and OPO laser excitation is schematized in the panel on the right, as well as the recorded signals (GFP, RFP, SHG, THG) and, when appropriate, the detected structure (e.g. collagen). (C) Harmonic signals revealing skin and skull morphology (blue on the 3D rendering of a thick optical cross section from an entire whole-mount imaging stack, left panel) are used to position the fish in an identical manner over the different imaging sessions. The aNSC layer (green, with processes extending into the brain parenchyma) is visible as a fluorescent signal underneath (left panel), or can be shown as a single whole-mount dorsal fluorescent recording (right panel). (D) The images recorded over consecutive sessions (repetitions of the sequence shown in A-C, e.g. here four times at a few days interval) are aligned using SHG/THG signals, followed by manual corrections using Imaris, to track individual cells over time. Dashed boxes outline the same group of cells recognizable over the consecutive time points. Scale bars: 50 μm (C), 15 μm (D).

To address these issues, we developed an experimental workflow (Fig. 1) permitting long-term observation of aNSCs ‘at work’ in their endogenous environment over days or weeks within the brain of the adult zebrafish. Our approach uses combined multicolor fluorescence (Mahou et al., 2012) and harmonics (Olivier et al., 2010) multiphoton microscopy and a homemade transparent transgenic line highlighting cell divisions. Fluorescence signals are used to detect aNSC markers, whereas endogenous second- and third-harmonic generation (SHG and THG, respectively) (Olivier et al., 2010) signals provide persistent morphological landmarks for longitudinal imaging on different days and for data registration. Using this approach, we show that it is possible to image aNSCs through the skin and skull of a live anesthetized animal every 2-4 days, and re-align large 3D images acquired over weeks. Image registration based on multiphoton signals then allows identification and tracking of thousands of aNSCs with single-cell resolution. This approach enabled us to characterize *in vivo* the distribution, frequency and mode of aNSC division events in their endogenous niche and across pallial areas.

## RESULTS

The zebrafish brain harbors widespread neurogenesis in several regions, mainly at ventricular surfaces (Adolf et al., 2006; Grandel et al., 2006). The dorsal telencephalon (pallium) is to date the best-studied neurogenic region under physiological conditions as well as in the context of post-injury regeneration or drug treatments (Kizil et al., 2012). The pallial germinal zone of the zebrafish is particularly relevant as it encompasses areas homologous to the two known constitutively active aNSC niches in rodents (Dirian et al., 2014). In addition, aNSCs identified in this territory are reminiscent of mammalian aNSCs (März et al., 2010). First, they are radial glial cells (RG) expressing markers such as Glial Fibrillary Acidic Protein (Gfap), Brain Lipid Basic Protein (Blbp; Fabp7a – Zebrafish Information Network), S100β or Glutamine Synthase (GS), and neural progenitor markers (Sox2, Nestin). Second, they are strongly quiescent, only ∼15% of them being activated at a given time point and expressing S/G2/M markers such as Mini-Chromosome Maintenance factor 5 (Mcm5) or Proliferating Cell Nuclear Antigen (Pcna). Third, they can undergo self-renewing symmetric or asymmetric divisions, the latter generating non-glial progenitors that will divide to generate neurons (Rothenaigner et al., 2011). Of high interest here, the pallial ventricular zone becomes exposed to the telencephalic surface following a morphogenetic process of eversion taking place during teleost embryogenesis (Folgueira et al., 2012). Thus, the cell bodies of adult RG are only separated from the brain surface by the thin tela choroida that is closing the ventricle. Based on the latter property, we hypothesized that it may be possible to image these cells directly in their endogenous environment using multiphoton (non-linear optical) microscopy.

To optimize tissue transparency, we selected *casper* transparent fish, which carry a double mutation for pigmentation genes (White et al., 2008) and have been successfully used for blood cell imaging in adults (Zhang et al., 2012). The choice of a *casper* background was made also to ensure reliable imaging, because the presence of pigments generally introduces absorption-mediated phototoxic routes that can hamper long-term multiphoton imaging (Débarre et al., 2014). Using double immunofluorescence staining for RG (GS) and proliferation (Pcna), we first verified that the *casper* pallial germinal zone harbors the expected proportions of the different neural progenitor subtypes (quiescent RG, activated RG and non-RG progenitors, respectively types 1, 2 and 3 cells) (März et al., 2010; Fig. S1), validating these mutants for the analysis of aNSCs. Next, to permit the live identification of pallial aNSCs, two transgenic reporter lines, *gfap:eGFP* (Bernardos and Raymond, 2006) and *her4:dRFP* (Yeo et al., 2007), were brought into the *casper* background. We previously showed that both lines reliably and specifically label aNSCs in the zebrafish pallium (Chapouton et al., 2010).

In order to guarantee fish survival, we designed a transitory anesthesia and observation procedure for repeated recordings over days and we minimized the duration of the imaging assay (Fig. 1A). Fish anesthesia was induced by immersion into a 0.02% tricaine (MS-222) solution for 90 s, then maintained by intubation and perfusion with 0.005% tricaine and 0.005% isoflurane (Fig. S2; Table S1). Fish were then mounted in a plastic dish and held between pieces of sponge. Adult zebrafish of 3 months of age or more consistently survived for at least 6 h in these conditions and recovered within a minute when transferred into their regular water.

For imaging, we used two synchronous excitation pulse trains at 820 nm and 1180 nm and wavelength mixing (Mahou et al., 2012) in order to detect several multiphoton signals simultaneously while ensuring image co-registration. We could then simultaneously detect two-photon excited fluorescence (2PEF) signals from RFP and GFP markers, second harmonic generation (SHG) from skull collagen, and third harmonic generation (THG) from skin cells and lipids. SHG/THG signals from the skin and skull above the telencephalon revealed characteristic structures such as cranial sutures (Fig. 1C; Figs S3, S4; Movies 1-3) that were used as global landmarks to find rapidly the previously imaged volume in the anesthetized fish at each experiment time point. The entire process of anesthesia, positioning, imaging and release typically took <45 min per fish, and guaranteed survival.

For each fish (*n*=6), we imaged a volume of 800 μm×800 μm×250 μm starting from the skin surface, with a voxel size of 0.83 μm laterally and 2 μm axially. Fluorescence and harmonic signals were recorded simultaneously and detected on separate detectors (Fig. S4; Fig. S5A,A′). Size, contrast and resolution of the fluorescence images were appropriate for visualizing all subdivisions of the pallial germinal zone, and for reaching a depth of 100-150 μm into the parenchyma. Such datasets encompassed a field of a minimum of 1000 RG with single-cell resolution (see below) and also provided a clear visualization of the radial basal processes in the dorsal pallial area (Dm, Dd), as seen in *xz* projections (Fig. S4B,C; Movies 1-3).

To monitor changes of the aNSC sheet and follow the behavior of individual cells over time, we imaged the same volume every 2-4 days in each animal and used spatial registration of imaging datasets to align the time points down to the cellular level. For this, we used a two-step procedure. First, we used the SHG/THG image data and automated pixel-based registration [RecursiveReg Matlab script for Imaris (Thévenaz et al., 1998); Fig. S5] to obtain coarse alignment of the brain images. Then, we manually refined the alignment of the 3D image sequences at the cellular level using landmark-based registration: a few cells were segmented and manually tracked over time and their average drift was corrected using Imaris to align small regions of the brain precisely (Fig. 1D; Fig. S6A,A′,C,C′). Following this alignment, we verified the accuracy of cell identification over time. For this, we calculated the 3D image cross-correlation between successive time points for 100 *her4:drfp*-positive cells randomly distributed within large germinal zone areas in two fishes [entire pallium in the fish named piwi, dorso-medial pallium (Dm) in the fish named fifi]. A cubic interrogation window of 40 µm per side was used. Cross-correlation values for tracked cells were significantly higher than when the same analysis was conducted after random permutations of cell positions (Fig. S6B, control; data not shown). Cross-correlation values remained high between two time points taken one month apart (cross-correlation between 11 randomly selected tracked cells in the fish named fifi; Fig. S6D), confirming that the pattern of fluorescent signal surrounding tracked cells matches well between successive time points, even when the delay is >30 days. In addition, this correlation analysis provides an estimation of the local error of image alignment by measuring the displacement of the correlation peak within the correlation volume: this error was systematically below the cell size, corresponding to the error on cell position estimation (5-10 µm, i.e. the average diameter of a RG cell). Together, this 3D cross-correlation analysis confirms that the image alignment is possible and successful even at the cellular level and that small groups of cells can be identified and tracked over weeks.

Image alignment makes it possible to determine whether individual or collective cell movements take place within the pallial germinal zone over time. To assess the degree of cell migration, we traced all imaged RG cells (*her4:drfp*-positive) across the entire germinal zone in piwi (>1100 cells at each time point; Fig. S7A-C′) and across three regions in piri (>370 cells; Fig. S7D,D′). Individual cell displacements over 10 days of tracking did not exceed, on average, one cell diameter (6.59 µm, s.d.=2.4, which is also near the range of precision errors in plotting cell centers). We did not detect individual ‘outlier’ cells with a different migration scale than others (Fig. S7B), and analysis of all displacement vectors did not reveal detectable collective movements at any site (Fig. S7C-D′). We conclude that pallial aNSCs are largely not migrating at time scales of 2-3 weeks and that the organization of the tissue stays very similar over time down to the scale of individual cells.

These data suggest that one should be able to follow the dynamics of the aNSC sheet at the level of single cell division events. However, no transgenic line is currently available to report a re-entry into the cell cycle, except for the Fucci line recently described (Bouldin and Kimelman, 2014), which has the limitation of not distinguishing G1 and G0 phases, thereby highlighting most aNSCs irrespective of their quiescent or activated status. To address this issue, we generated a transgenic line expressing eGFP in activated cells using conserved 5′-flanking regions of the *mcm5* gene (Fig. S8). Expression of *mcm3/3/5/7* genes is downregulated, at the transcriptional level, in most mammalian cells and tissues in G0 (Stoeber et al., 2001) and, in zebrafish, *mcm5* transcription closely matches *pcna* transcription in the embryonic retina and is absent from quiescent cells in this tissue e.g. in Müller glial cells (Ryu et al., 2005). We verified the reliability of this line until adulthood by double immunocytochemistry comparing GFP and Mcm5 protein, or GFP and Pcna (Fig. S9), and showed highly similar patterns in pallial RG.

**Fig. 2.**
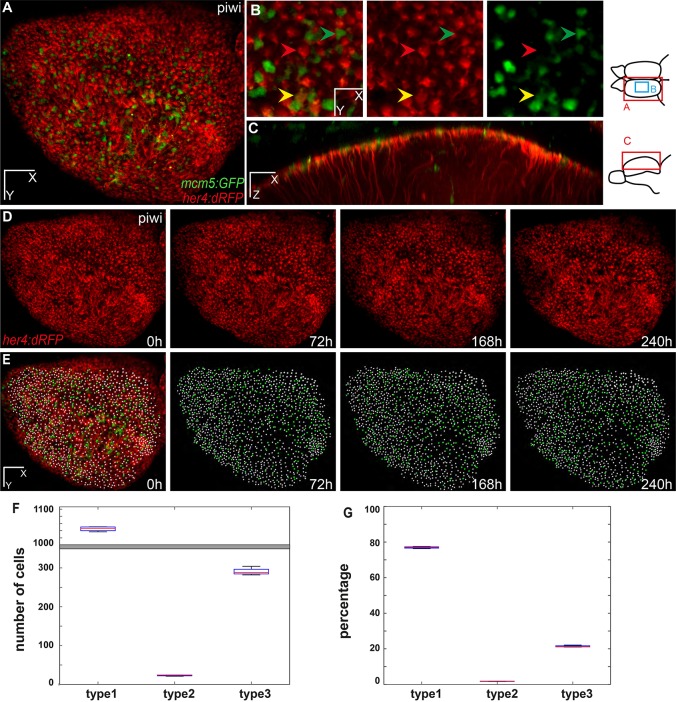
**Live imaging and tracking over 10 days of all adult neural stem cells across the entire pallial germinal zone.** (A-C) One hemisphere of a *her4:dRFP;mcm5:eGFP;casper* transgenic fish (individual fish named piwi) imaged through the skin and skull. Anterior to the left. (A) Dorsal view of an entire hemisphere. (B) High magnification of a central area of the medial pallium, observed live, highlighting examples of quiescent RG (type 1), proliferating RG (type 2) and proliferating non-glial progenitors (type 3 cells) (red, yellow and green arrowheads, respectively). (C) Optical sagittal section showing the processes of the glial cells through the parenchyma. Diagrams to the right show the regions imaged for A,B and C. (D,E) Dorsal views of the pallial germinal zone in the same animal after alignment over 10 days of imaging. (D) Red channel highlighting all RG cells. (E) RG cells (white dots; 1122 cells on average, s.d.=13) and proliferating cells (green dots; 290 cells on average, s.d.=9) plotted over time across the entire germinal zone (superimposed to the fluorescence image on the left panel). (F,G) Box plot of the total number (F) and percentage (G) of progenitor cells of each type, averaged over the different time points of imaging. The mean number of type 1 cells counted is 1042 (s.d.=7.6), of type 2 cells is 91 (s.d.=6.6) and of type 3 cells is 391 (s.d.=9.5). The percentages of type 1, 2 and 3 cells over all cells (type 1+2+3) are 73.5%, 5.7% and 20.6%, respectively. Scale bars: 80 μm (A,D,E), 30 μm (B), 50 μm (C).

For live aNSC imaging, the *mcm5:eGFP* line was bred into the *her4:dRFP;casper* transgenic double mutant background, and fish were imaged repeatedly at regular intervals over a period of 2 weeks as described above. We could detect on average ∼1500 dRFP-positive aNSCs and 400 proliferating eGFP-positive cells per brain per time point (Fig. 2A-E; Fig. S10; Movie 4), which is consistent with the proportion of RG and activated progenitors described in the literature (März et al., 2010) (see also Fig. S1). Importantly, and as expected, these values appeared to be constant over time (Fig. 2F,G), stressing the reliability of our transgenes and of our imaging methodology to reveal the bona fide status of the endogenous pallial niche at any given time point.

**Fig. 3.**
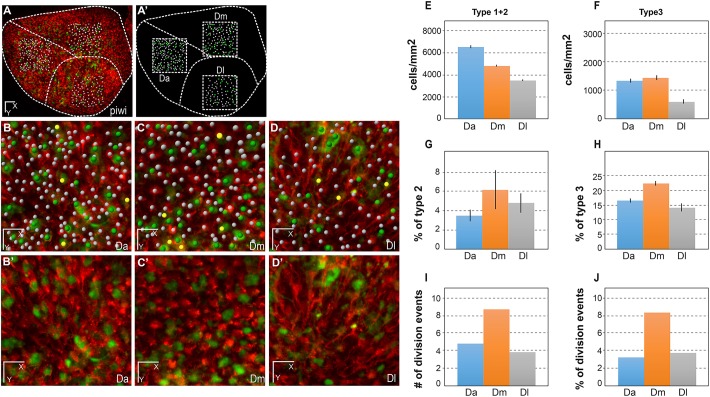
**Quantitative analysis of dynamic NSC parameters in distinct domains of the pallial germinal zone (Da, Dm and Dl) in the fish named piwi.** (A,A′) Live image of the pallial germinal zone showing *her4:drfp-*positive NSCs (red) and *mcm5:egfp* dividing cells (green) (A) and positioning of the three sampled subdomains (dashed squares representing a top view of the sampled cubes) (A′). Dashed lines separate the corresponding neuroanatomical domains. Anterior to the left, midline to the top. Da, anterior pallium; Dm, medial pallium; Dl, lateral pallium. Adapted from Wullimann et al. (1996). (B-D′) High magnification views of the three domains, as indicated, with (B-D) and without (B′-D′) individual cell plotting. White dots, quiescent NSCs; yellow dots, activated NSCs; green dots, dividing *her4-drfp*-negative progenitors. (E-H) Quantification of NSC parameters over 10 days (t1-t4). All statistics are paired *t*-tests, with standard deviations indicated as vertical bars. The average numbers of NSCs counted for each domain per time point are 151 (Da), 109 (Dm) and 83 (Dl). (E) NSC density in each domain, measured as the total number of type 1+type 2 cells per mm^2^ of germinal zone surface (see Materials and Methods for the calculation of this surface taking into account the germinal zone curvature). Da versus Dm: *P*<0.001; Da versus Dl: *P*<0.0001; Dm versus Dl: *P*<0.0001. (F) Density of type 3 cells (non-glial proliferating progenitors), measured as the total number of type 3 cells per mm^2^. Da versus Dm: *P*<0.1; Da versus Dl: *P*<0.0001; Dm versus Dl: *P*<0.0001. (G) Proportion of activated NSCs (type 2 cells) in each domain among the total NSC population (type 1+type 2 cells). Da versus Dm: *P*<0.1; Da versus Dl: *P*<0.05; Dm versus Dl: not significant. (H) Proportion of type 3 cells among the total progenitor population (type 1+type 2+type 3 cells). Da versus Dm: *P*<0.001; Da versus Dl: *P*<0.05; Dm versus Dl: *P*<0.01. (I,J) Quantification of *de novo* NSC activation events over 7 days (t2-t4) in absolute numbers (I) and in proportion of the number of RG quiescent at time t4 (J). Da versus Dm: *P*=0.1 (not quite significant); Da versus Dl: *P*=0.7, not significant; Dm versus Dl: *P*=0.5 (not significant); Fisher's two-tailed test. Scale bars: 50 μm (A,A′), 30 μm (B-D′).

To date, the characteristics and division properties of adult pallial NSCs have been inferred by analyzing restricted areas on 2D tissue sections at a given time point, or, across the pallium and over weeks, following a few tracked cells labeled by lentiviral transduction or electroporation (Barbosa et al., 2015; Rothenaigner et al., 2011). The imaging methodology reported here, and the *mcm5:eGFP* line, make it possible for the first time to extract basic parameters describing the behavior of the entire pallial NSC population in time and space, and to determine whether distinct neuroanatomical areas differ in these properties. To do this, we used analysis regions of 150 µm per side in three distinct domains: the anterior part of the pallium (Da), the dorso-medial pallium (Dm) and the lateral pallium (Dl) (Fig. 3A,A′). These areas differ in neuroanatomical organization, lineage and function (Dm and Dl, for example, have been proposed to host amygdalar and hippocampal functions, respectively; Dirian and Bally-Cuif, 2014; von Trotha et al., 2014; Wullimann et al., 1996). We plotted all cell types in these domains in two fish (Fig. 3A-D; Fig. S11A-C). Quantifications from imaging conducted at regular intervals (t1=0 h, t2=72 h, t3=168 h, t4=240 h) over 10 days showed that the number of NSCs (type 1+type 2 RG cells) per surface area (NSC density) was higher in Da than in the other domains (Fig. 3E; Fig. S11D). Da, compared with Dm and Dl, was also characterized by a lower percentage of activated NSCs among the NSC population, in a constant manner over the 10 days of analysis (Fig. 3G; Fig. S11F). Likewise, the proportion of type 3 progenitors (proliferating non-glial progenitors) among the entire progenitor population (type 1+type 2+type 3 cells) was lower in Da (Fig. 3F,H; Fig. S11G). Showing similar correlations, Dm harbored, compared with Dl, higher NSC density and a lower percentage of type 3 cells. However, the proportion of type 2 cells within the NSC population was either not significantly different between the two domains or slightly higher in Dm (Fig. 3E-H; Fig. S11F). Because GFP is a fluorescent tracer that appears to be stable for a few days in the context of active NSCs and their progeny (like immunocytochemical markers such as Gfap or Mcm proteins), we relied on the number of cell division events initiated after the first imaging time point to assess quantitatively the dynamics of NSC activation in the three domains. Quantification of activation events over 7 days (i.e. RG cells newly switching on GFP at time points t2 to t4) revealed that activation was more frequent in Dm than in the two other domains, both in absolute values and in the proportion of quiescent NSCs at time t4 (trend in one fish, significant in the other; Fig. 3I,J and Fig. S11H,I, respectively). Dl exhibited medium levels of *de novo* activation, and the lowest levels occurred in Da. These results validate our transgenic combination for imaging the slow dynamics of the aNSC sheet, and highlight the existence of pronounced regional differences in NSC dynamics across the NSC population.

**Fig. 4.**
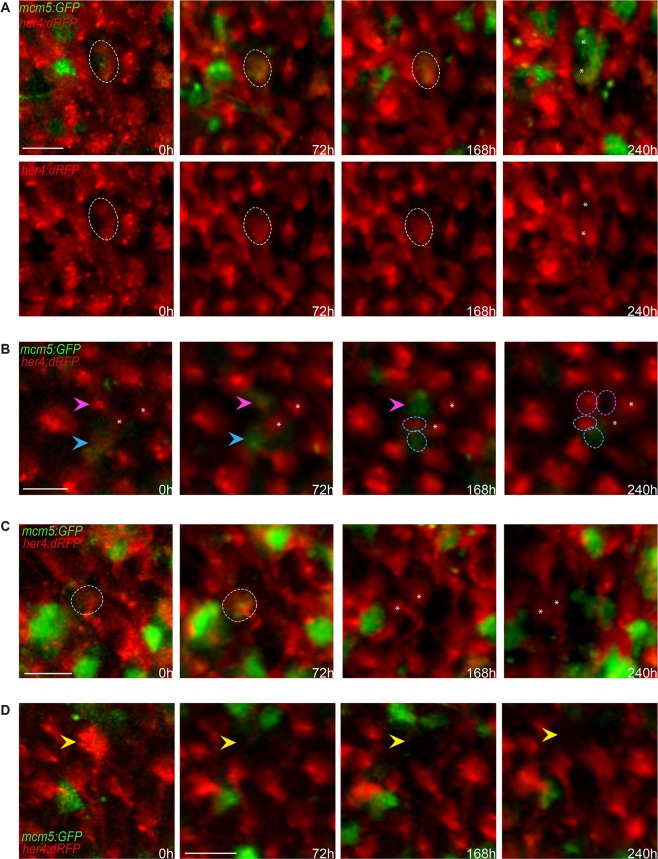
**Qualitative evaluation of NSC fate over 10 days across the entire pallial germinal zone.** (A-D) Detailed views (dorsal) of groups of cells in a *her4:dRFP;mcm5:eGFP;casper* transgenic fish (individual fish named piwi) observed *in vivo* through the skin and skull over four time points (t1-t4) spanning 10 days. (A) Example of a gliogenic (self-renewing) division with asymmetrical outcome at time t4. The circle outlines an RG cell expressing RFP and GFP on the first and following days, which divided at t4 to give rise to two daughter cells (asterisks) that maintained GFP expression but only one of which (bottom cell) expresses RFP (and harbors a process, not shown). Top panel: merged channels; bottom panels: red channel only. (B) Examples of two other gliogenic (self-renewing) divisions with asymmetrical outcome at time t4. The magenta arrowhead indicates an RG cell expressing RFP on the first day, switching on GFP at t2 and dividing at t4 to give rise to two daughter cells (circles), both non-proliferating, one expressing RFP (and harboring a process, not shown). The cyan arrowhead indicates an RG cell expressing RFP and GFP on the first and following days and dividing at t4 to give rise to two daughter cells (circles), one expressing GFP and the other RFP (and harboring a process, not shown). Asterisks indicate two quiescent cells that serve as references. (C) Example of a symmetric gliogenic division. The circle indicates an RG cell expressing RFP on the first day, then switching on GFP and dividing to give rise to two identical RFP-positive, GFP-negative (quiescent) daughter cells (asterisks). (D) Example of an RFP-positive RG cell (yellow arrowhead) losing RFP expression over the time course of analysis; this is interpreted as the cell switching fate and delaminating into the parenchyme. Scale bars: 20 μm (A-D).

Our imaging approach also permits assessing division modes and NSC fates across the pallial germinal zone. Most NSCs remained quiescent during the 10 days of recording (>90% of the cells, largely outnumbering all other behaviors) (Fig. 3J; Fig. S11I, no activation in >90% of the tracked NSCs; Table S2, case 1). We note that the slow speed of division events, and the tendency for *her4:drfp* expression to decrease or even completely fade during division, left some division events unresolved over the 10 days of tracking (Table S2, cases 2-3; Fig. S12A). Among the complete division or fate events that we could track, we could record four distinct NSC behaviors during a 10-day period: (1) self-renewing divisions with an asymmetry in the proliferation status and/or identity of daughter cells at time t4 (gliogenic division of a *her4:dRFP*-positive RG into a *her4:dRFP*-positive RG, proliferating or not, and a *her4:dRFP*-negative cell, proliferating or not) (Fig. 4A,B; Fig. S12C; Table S2, cases 7-8), (2) self-renewing, symmetric gliogenic divisions giving rise to two quiescent NSCs (symmetric gliogenic division of a *her4:dRFP*-positive RG into two *her4:dRFP*-positive RG) (Fig. 4C;Table S2, case 6), (3) symmetric divisions giving rise to two daughters that do not express *her4:dRFP* at time t4 (Fig. S12D; Table S2, case 8), and (4) NSCs ‘disappearing’ without a division event (Fig. 4D; Table S2, case 10). We interpret the latter fate (NSC ‘loss’, *her4:dRFP*-positive RGs disappearing from the field of view within 72 h and not re-expressing *her4:dRFP* for at least 7 days) as RG cells that ceased to express *her4* without dividing (no *mcm5:eGFP* expression was detected), suggesting that they may have directly transformed into another cell type. We consider it less likely that the cell died, given that apoptosis is extremely rare in the adult zebrafish telencephalon (Webb et al., 2009) and that we did not visualize cell debris. From previous tracing studies, the fate change is likely to be towards the direct generation of a neuron, diving into the parenchyme (Barbosa et al., 2015). However, we also cannot exclude the contribution to this category of some *her4:drfp*-positive RG transiting into another NSC state not expressing *her4*. Slow fluctuations of *her4*, or heterogeneity within the NSC population with respect to *her4* expression, might account for this. Generally, we remain cautious regarding the interpretation of all divisions giving rise to a *her4:drfp*-negative daughter (Table S2, cases 7-9), as it is possible that this daughter cell is committed to a non-glial amplifying fate (e.g. a type 3 progenitor) or to re-express *her4* at a later time point not covered by our imaging series [see also Table S2, case 5, although this appears to be an extremely rare event (1 in 107 recorded state change events)]. The number of recorded cells in each case is too low to conduct meaningful statistical analyses comparing our data between pallial domains and with the existing literature; in addition, these studies have used different parameters to record RG cells (Gfap or GS expression in a study by Rothenaigner et al., presence of a radial process in a study by Barbosa et al., and *her4:drfp* expression in the present work, as we could observe radial processes re-growing from red cell bodies following divisions). However, qualitatively, our data confirm the division modes and fates observed in the previous tracing studies (Barbosa et al., 2015; Rothenaigner et al., 2011).

## DISCUSSION

**Fig. 5.**
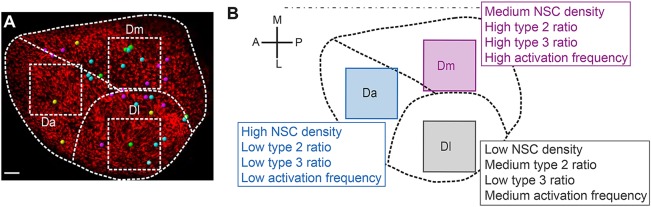
**Schematic summary of the distinct NSC parameters observed across the pallial germinal zone.** (A) Spatial distribution of the different types of division events completed over the imaging period (Table S2, cases 6 to 9) among 1138 tracked cells (from Fig. 4, Fig. S12). Yellow dots, symmetric gliogenic symmetric divisions; cyan dots, asymmetric gliogenic generating a RG and a ‘lost’ cell at t4; magenta dots, asymmetric gliogenic generating a RG and a proliferating progenitor at t4; green dots, symmetric with an undetermined fate of both daughters. These correspond respectively to cases 6 to 9 in Table S2. (B) Cell density and activation frequency parameters mapped in Da, Dm and Dl (from Figs 2 and 3 and Fig. S11). In both panels, one hemisphere is shown, anterior to the left. Scale bar: 50 μm.

Together, the optical method reported here, combining harmonics and fluorescence recording, and the transgenic/mutant background we developed, establish that aNSCs can be dynamically imaged and tracked as an entire germinal population of >1000 cells and at single-cell resolution in their endogenous niche in the adult vertebrate brain of a living animal over the course of several weeks. Importantly, this makes it possible to capture the slow dynamics of adult neural germinal zones in their native environment, and to describe the spatiotemporal distribution and coordination of aNSC activation events at the level of an entire NSC population (Fig. 5A,B). We note that the resolution achieved down to a depth of 100 µm inside the tissue would make it possible to monitor the generation of neurons, by using an appropriate transgenic background. Alternatively, Barbosa et al. recently used live confocal imaging to follow electroporated cells in a uniformly labeled *gfap:egfp* transgenic NSC sheet and highlight distinct NSC division modes triggered by mechanical lesion (Barbosa et al., 2015). Because a number of small molecules affecting adult neurogenesis cross the blood-brain barrier and can be simply applied to fish in the swimming water, this methodology also allows the possibility of directly monitoring the effect of pharmacological treatments of the aNSC sheet in real time. In both cases, however, a key aspect will be to take into account the relative behavior of neighboring cells and the spatial localization of the traced NSCs. Indeed, an important finding of the present work is that distinct pallial domains harbor NSCs that differ in several parameters, including NSC density and activation frequency. Our imaging, alignment and tracking methodology, which allows a large cell population spanning these different domains to be followed, will permit such comprehensive analyses. Whether there is a causal relationship between the different NSC parameters that exhibit regional variation also remains an interesting aspect to study in order to understand NSC dynamics.

## MATERIALS AND METHODS

### Fish husbandry and lines

Zebrafish (*Danio rerio*) were kept at 28.5°C, pH 7.4 and salinity-controlled conditions. All experiments were carried out in accordance to the official regulatory standards of the Department of Essonne (agreement number A 91-577 to L.B.-C.). Three- to four-month-old Tg(*gfap:eGFP*) (Bernardos and Raymond, 2006) (referred to as *gfap:gfp*), Tg(*her4:dRFP*) (Yeo et al., 2007) (referred to as *her4:dRFP*) and Tg(*mcm5:eGFP*)^gy2^ (referred to as *mcm5:gfp*) (this study) zebrafish in the *casper* double mutant background (*roy^−/−^;nacre^−/−^*) (White et al., 2008) were used in all experiments.

### Generation of the *Tg(mcm5:egfp)^gy2^* transgenic line

This line carries a 1200 bp *mcm5* enhancer region fused to *egfp*. See supplementary materials and methods for further details.

### Immunohistochemistry

Dissected brains were fixed overnight in 4% paraformaldehyde in PBS and kept in 100% methanol at −20°C. Following rehydration and vibratome sectioning at 50 μm thickness, the following primary antibodies were used: chicken anti-GFP (1:500, Aves Labs, GFP-1020), mouse anti-glutamine-synthase (1:500, Millipore, MAB302), rabbit anti-PCNA (1:250; Tebu-Bio, GTX124496). Goat antibodies coupled to AlexaFluor dyes (488, 555 or 647; Invitrogen) were used as secondary antibodies. Images were taken using a confocal microscope (LSM700, Zeiss) and analyzed with Imaris 7 (Bitplane).

### Fish anesthesia and mounting for imaging

Anesthesia was induced with 0.02% MS22 (Sigma) and maintained with 0.005% MS222, 0.005% isoflurane. See supplementary materials and methods for further details.

### Multiphoton microscopy

Combined multicolor two-photon/SHG/THG imaging was performed on a custom-built point-scanning upright microscope and the wavelength mixing method is described by Mahou et al. (2012).

### Experimental conditions for live GFP/RFP/harmonics imaging

For simultaneous excitation of these signals, titanium:sapphire (TiS), optical parametric oscillator (OPO) and two-color equivalent excitation λ_3_=2/(1/λ_TiS_+1/λ_OPO_) wavelengths were set to 820 nm, 1180 nm and 968 nm, respectively. RFP excitation was obtained mainly from the OPO beam and detected on a ‘red’ channel; GFP excitation was obtained through a two-color two-photon process and detected on a ‘green’ channel; THG signals from 1180 nm excitation and SHG signals from 820 nm excitation were simultaneously detected on the same ‘blue’ channel. We point out that with this type of excitation, dense non-centrosymmetric structures, such as fibrillar collagen, produce signals at three wavelengths: SHG from both excitation beams at 420 nm and 590 nm, and sum-frequency generation (SFG) at 484 nm. The pixel dwell time was 5 µs, and the voxel size was 0.8×0.8×2 µm^3^.

### Experimental conditions for live THG/SHG imaging

For the THG/SHG mosaic shown in Fig. S3 and Movies 1-3, we used only one excitation wavelength (λ_OPO_=1180 nm), and we detected THG signals λ/3 and SHG signals λ/2 on separate channels. The voxel size was 2×2×2 µm^3^.

### Image alignment and analyses

The *z*-stacks acquired on successive imaging sessions were first converted into a single file after cropping two files in the three dimensions using Imaris (Bitplane) or Fiji. To obtain a coarse alignment of the brain images using the harmonic signals only and the pixel-based registration of SHG/THG image sequences, we used the RecursiveReg Matlab script for Imaris provided by Michael Liebling (UCSB, California, USA) (Thévenaz et al., 1998) without allowing deformation (‘rigid body’ option). The alignment was refined at the cellular level using landmark-based registration for which a few cells are segmented and manually tracked over time and their average drift was corrected using Imaris. For Fig. S4, the green signal from the GFP was very strong, so we subtracted the green signal from the SHG/THG channel and adjusted the histograms. For all the time-lapse images and movies we adjusted the intensity by eye (linear stretch of the histograms) to correct the minor fluctuations in intensity from one day to another.

After alignment, all cells were automatically segmented. RFP cells were semi-automatically tracked using Imaris. RG divisions were manually identified.

Regions of interest (ROI) were defined in Da, Dm and Dl. Surface areas were calculated using the irregular quadrilateral area formula and the ‘real’ length of the ROI to account for the curvature of the surfaces. Densities were then calculated by dividing the number of cells by the area.

### Image cross-correlation analyses

3D image cross-correlation was computed using the ‘normxcor3’ Matlab function. See supplementary materials and methods for further details.
